# The sintering temperature effect on electrochemical properties of Ce_0.8_Sm_0.05_Ca_0.15_O_2-δ_ (SCDC)-La_0.6_Sr_0.4_Co_0.2_Fe_0.8_O_3-δ_ (LSCF) heterostructure pellet

**DOI:** 10.1186/s11671-019-2979-x

**Published:** 2019-05-14

**Authors:** Xiyu Nie, Ying Chen, Naveed Mushtaq, Sajid Rauf, Baoyuan Wang, Wenjing Dong, Xunying Wang, Hao Wang, Bin Zhu

**Affiliations:** Key Laboratory of Ferro and Piezoelectric Materials and Devices, Faculty of Physics and Electronic Science, Hubei University, Youyi Road 368, Wuhan, 430062 Hubei People’s Republic of China

**Keywords:** SOFC, Semiconductor-ionic materials, Sintering temperature, EFFC, Heterostructure-semiconductor, Interfacial ion-conduction

## Abstract

Recently, semiconductor-ionic materials (SIMs) have emerged as new functional materials, which possessed high ionic conductivity with successful applications as the electrolyte in advanced low-temperature solid oxide fuel cells (LT-SOFCs). In order to reveal the ion-conducting mechanism in SIM, a typical SIM pellet consisted of semiconductor La_0.6_Sr_0.4_Co_0.2_Fe_0.8_O_3-δ_ (LSCF) and ionic conductor Sm and Ca Co-doped ceria Ce_0.8_Sm_0.05_Ca_0.15_O_2-δ_ (SCDC) are suffered from sintering at different temperatures. It has been found that the performance of LSCF-SCDC electrolyte fuel cell decreases with the sintering temperature, the cell assembled from LSCF-SCDC pellet sintered at 600 °C exhibits a peak power density (*P*_max_) of 543 mW/cm^2^ at 550 °C and also excellent performance of 312 mW/cm^2^ even at LT (500 °C). On the contrary, devices based on 1000 °C pellet presented a poor *P*_max_ of 106 mW/cm^2^. The performance difference may result from the diverse ionic conductivity of SIM pellet through different temperatures sintering. The high-temperature sintering could severely destroy the interface between SCDC and LSCF, which provide fast transport pathways for oxygen ions conduction. Such phenomenon provides direct and strong evidence for the interfacial conduction in LSCF-SCDC SIMs.

**Electronic supplementary material:**

The online version of this article (10.1186/s11671-019-2979-x) contains supplementary material, which is available to authorized users.

## Introduction

Recently, hydrogen as promising clear energy has attracted extensively interesting [1, 2]. Solid oxide fuel cell (SOFC) can directly convert the chemical energy of hydrogen into electricity without burn and presented immense potential in the future energy market due to its unique advantages, including high efficiency, fuel flexibility, and environmental friendliness [3, 4]. However, there are several bottlenecks associated with conventional SOFC, one of the major challenges is high operational temperature, which still plagued many researchers. The yttrium-stabilized zirconia (YSZ) and doped ceria as the desired electrolyte materials should fulfill sufficient ion-conducting at more than 800 °C [5, 6]. High operational temperature needs an exact thermal match and chemical compatibility between anode, cathode, and electrolyte. The other challenge for conventional SOFC is the high sintering temperature (> 1000 °C), which is the essential condition for obtaining dense electrolyte layer in order to prevent the direct interaction between fuel and air [7]. The dense electrolytes have great mechanical strength with enhanced physical stability at high temperature or other harsh conditions. Both high operating temperature and high sintering temperature will definitely induce extensive costs and seriously hinder the commercialization of SOFC. To address these issues, Liu et al. have developed electrolyte-free fuel cell (EFFC) based on semiconductor and ionic materials (SIMs) [8], which usually consisted of semiconductor and ionic conductor heterostructure and delivered super-high ionic conductivity even at low temperature (LT). Barriocanal et al. reported that the SrTiO_3_-YSZ SIM displayed eight orders of magnitude enhancement for ionic conductivity in comparison with pure YSZ [9]. Yang et al. prepared vertical SIM nanocolumns consisting of samarium-doped ceria (SDC) and SrTiO3; its ionic conductivity is higher by one order of magnitude than plain SDC films [10]. In the Ce_0.8_Gd_0.2_O_2-δ_-CoFe_2_O_4_ SIM, it was found that the oxygen ion superiorly conducted along the grain boundary [11]. Due to the inherent high ionic conductivity, many SIMs have been utilized as an ion-conducting membrane to assemble the EFFCs, which presented high performance, such as 500–1000 mW/cm^2^ maximum power at operational temperatures below 600 °C [12–15]. Besides, our previous reports revealed that the SIMs has not been subjected to a high-temperature sintering process. The enhancement of conductivity in these SIMs was suggested to cause by the formation of a space charge region and a structural misfit at the interface region. Along these lines, the interface between two-phase materials dominated the conduction of charges which can be considered to be the effect of the composite. Actually, the sintering temperature is crucial for interface formation; high-temperature sintering can engender the non-uniform agglomerations and largely eliminate the interface area further to decrease the conductivity. On the other side, the LT sintering generated or produced pores structure in SIM layer and deteriorated the fuel cell performance. Therefore, the investigation of sintering temperature and the corresponding influence mechanisms toward fuel cell performance and further optimization are prerequisites for EFFCs.

In this work, LSCF-SCDC composite powders were suffered from sintering at different temperatures. The microstructure and morphology of LSCF-SCDC powders were detected by SEM images and XRD analysis, respectively. For practical application, the sintered powders were fabricated into EFFC devices for electrochemical measurements. Electrochemical impedance spectroscopy technique and electrical conductivity were utilized to explore the conductivity mechanism.

## Method

### Materials synthesis

Ion-conducting material Sm and Ca co-doped cerium oxide Ce_0.8_Sm_0.05_Ca_0.15_O_2-δ_ (SCDC) was synthesized by one-step co-precipitation method. According to the stoichiometry, certain amount of cerium nitrate hexahydrate (Ce(NO_3_)_2_·6H_2_O), samarium nitrate hexahydrate (Sm(NO_3_)_2_·6H_2_O), and calcium nitrate tetrahydrate (Ca(NO_3_)_2_·4H_2_O) were dissolved into deionized water to form a 1-M solution. In the meantime, 1 M of sodium carbonate aqueous solution was prepared and used as a precipitant; the ratio of metal ions to carbonate ions is 1:1.5. The above mixture of nitrate hydrate solution was gradually dropped into sodium carbonate solution at a rate of 10 ml/min during continuous stirring and white precipitates occur. Afterward, the resulting precipitates were filtered and washed with deionized water several times and dried in an oven at 120 °C for 10–12 h. Finally, the dried precursors were obtained and then calcinanted at 800 °C for 4 h. The obtained final product was fully ground to obtain yellowish powders for further usage. LSCF is purchased from Ningbo SOFCMAN Energy Technology Co., Ltd (China) as a commercial product. A series of LSCF-SCDC cells (40%:60%) was pressed at 220 Mpa and sintered at different temperatures. The diameter of the resultant LSCF-SCDC pellets is 13 mm and the thickness is about1.2 mm. The cells were sintering in a stagnant air at four different temperatures, i.e., 600, 800, 900, and 1000 °C for 10 h with a temperature increase rate of 10 °C/min.

### Microstructural characterization

The crystal structures of LSCF, SCDC, and LSCF-SCDC composites were characterized using a Bruker D8 X-ray diffractometer (XRD, Germany, Bruker Corporation) with Cu Ka (*λ* = 1.54060 A) radiation. The morphology of the samples was analyzed by a field emission scanning electron microscope (FESEM, JEOL JSM7100F Japan) equipped with an Oxford energy-dispersive spectrometer (EDS).

### Fuel cell fabrication and performance test

The fuel cell devices were fabricated, using NCAL powder (Tianjin Baomo Joint Hi-Tech venture) to prepare the slurry with terpineol and pasted on the nickel foam to form the Ni-NCAL layer. The prepared Ni-NCAL layer was dried at 120 °C for 15 min to evaporate the terpineol. The LSCF-SCDC cells were sandwiched between Ni-NCAL layers into fuel cell testing devices to measure the electrochemical properties. All fuel cells were subjected to a preheating treatment at 550 °C for 1 h. Hydrogen was supplied as the fuel at a flow rate of 80–120 ml/min, and the air was supplied as oxidant at 150–200 ml/min under 1 atm. The current-voltage and current-power curves of the fuel cells were recorded by a computerized instrument (ITECH8511, ITECH Electrical Co, Ltd).

### Conductivity measurements

LSCF-SCDC pellets sintered at different temperatures were painted with Ag paste on both sides, following heat treatment at 550 °C for 1 h, and then fixed on test holder for electrochemical impedance spectroscopy (EIS) measurements. The measurements were conducted by an electrochemical workstation (Gamry instrument reference 3000) under the open circuit mode with a 10-mV alternating current signal over the frequency range of 0.1–10^6^ Hz. The EIS results were simulated by Zsimwin software.

## Results and discussion

### Crystalline structure analysis

**Fig. 1:**
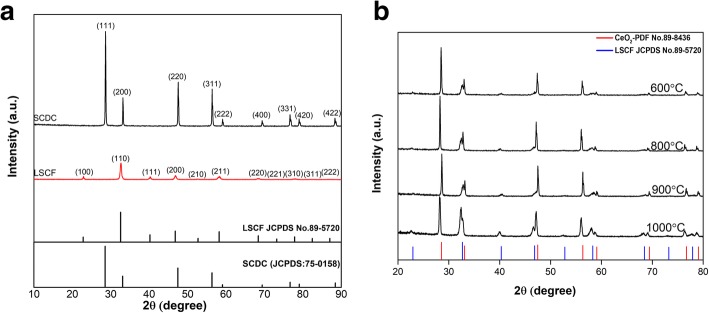
**a** The XRD patterns of SCDC and LSCF and their corresponding PDF card. **b** LSCF-SCDC samples sintered at different temperatures and pure CeO2 PDF card provided

The crystal structures of SCDC, LSCF, and their corresponding PDF card are depicted in Fig. 1a, The XRD pattern of SCDC was indexed to be cubic fluorite phase of SCDC (JCPDS 75-0158), which is highly similar with pure CeO_2_ (JCPDS 34-0394) [16] and the diffraction peak of SCDC presented slightly shifting towards the lower 2θ values in comparison with pure CeO_2_, indicating that both Sm and Ca are well doped into the crystal lattice of ceria, and the lattice constant has been enlarged after dual ion-doping according to Scherrer equation. The strong altitude of peaks demonstrated the high crystallinity of as-synthesized SCDC powder. For the LSCF XRD pattern, eleven diffraction peaks can be detected at 22.939°, 32.665°, 40.291°, 46.867°, 52.799°, 58.296°, 68.446° ,73.243°, 77.923°, 82.522°, and 87.073°,which could be indexed as (100), (110), (111), (200), (210), (211), (220), (221), (310), (311), and (222) planes respectively. The LSCF can be identified as pure perovskite structure and these results are consistent with the previous reported [17]. The XRD patterns for samples sintered at different temperatures are presented in Fig. 1b for comparison. It can be seen that the peak intensity decreased with annealing temperature increasing, and it is may be due to the degradation of LSCF into fine-grained Sr-O product at high temperatures. In the meantime, we can observe that the peaks position of LSCF-SCDC composite shifted toward small angle, and the crystal phase slight solubility between the LSCF and SCDC during sintering resulted in lattice expansion along with increasing the lattice constant [18], finally leading to XRD diffraction peaks shifts to a low angle. The interesting phenomena are the peaks of 900 °C pellet shift to high angle, and it is may be due to precipitation of Sr and Co caused by the LSCF degradation when the sintering temperature reached as high as 900 °C, which is well consistent with the previous literature [19]. As the temperature continually increases to 1000 °C, the grains kept on growing up, and the corresponding lattice constant is greater than that of 600 °C and 800 °C pellets, so it can be seen that the XRD peak moved back to a small angle. Moreover, the degradation is only a small amount of Sr and Co so that no independent peaks of Sr and Co were found. Almost all the characteristic peaks of SCDC and LSCF could be observed individually and no extra phase was detected, which certified that no chemical reaction occurred between LSCF and SCDC materials during the sintering processes even at 1000 °C. In other words, the LSCF-SCDC composite was relatively stable under the high temperature; the stability of materials is critical and a precondition for the stability of assembled fuel cells.

### Morphological characterization

**Fig. 2:**
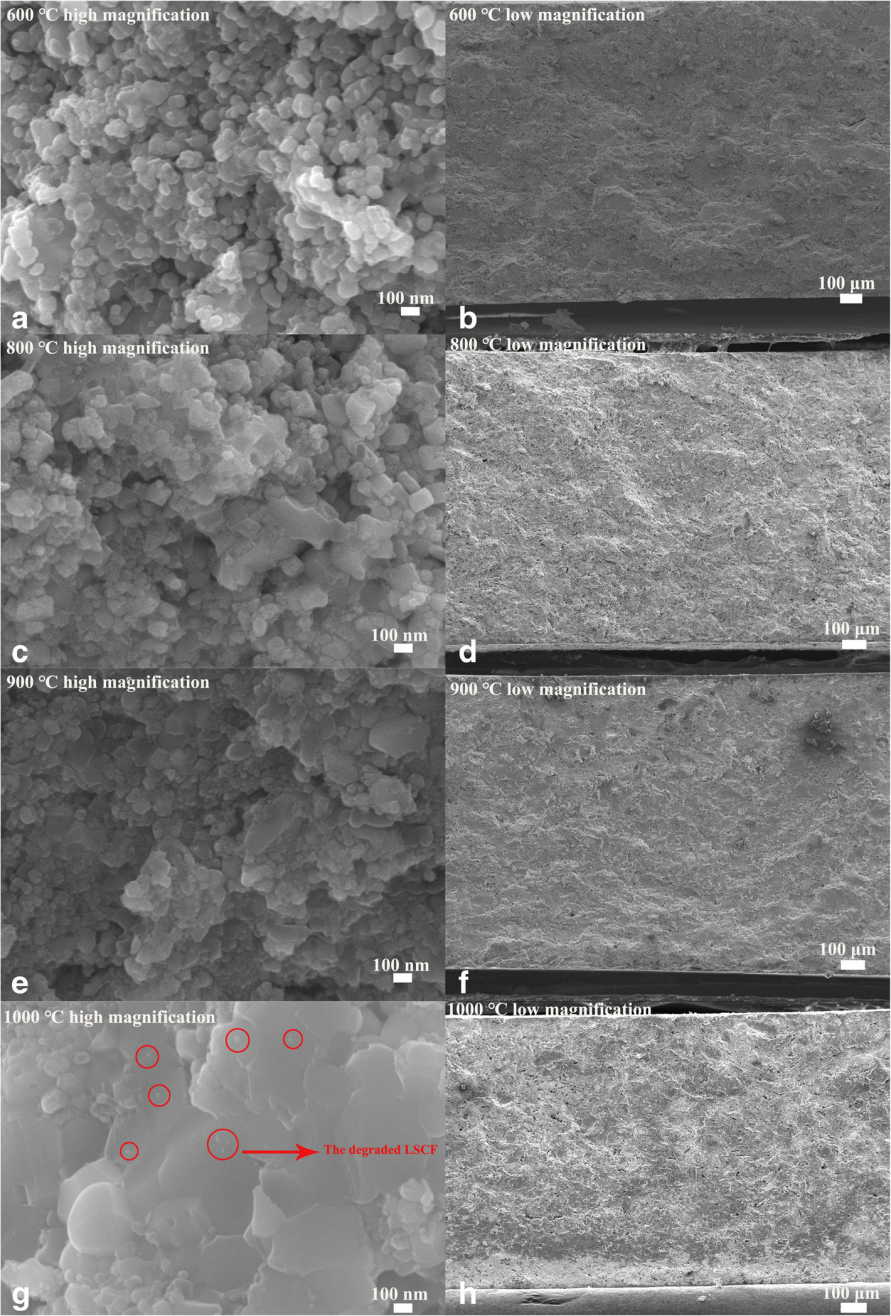
The cross-sectional SEM images in both high and low magnification of the LSCF-SCDC pellets sintered at different temperatures. (**a**, **b**) 600 ºC; (**c**, **d**) 800 ºC; (**e**, **f**) 900 ºC; (**g**, **h**) 1000 ºC

The cross-sectional SEM images of the LSCF-SCDC pellet sintered at different temperatures are shown in Fig. 2. As the high-magnification image Fig. 2a for 600 °C pellet shows, the sample consists of well-necked particles with a wide size distribution from nano-size to micro-size. It may be due to the use of commercial LSCF materials without elaborate control of particle size and morphology [20], as the SEM images for pure LSCF and SCDC are shown in Additional file 1. However, some agglomeration can be observed in pellets sintered at 800 °C and 900 °C. In the granular growth for the LSCF-SCDC pellet after high-temperature sintered at 1000 °C, particle shape has been badly destroyed to form larger clusters, which results in a significant decrease of the specific area. On the other hand, the pellet has also formed a bulk structure with higher density compared to pellets sintered at 600 °C. High-temperature sintering has eliminated the particle interfaces that can provide transport pathway for ionic conduction [21]. Obviously, the pellet thickness decreases with the sintering temperatures due to the shrinkage, and such phenomena generally occurred during the high-temperature sintering [22, 23]. Moreover, it can be seen that the density of the LSCF-SCDC pellet has gradually enhanced with the sintering temperature. To obtain the accurate thickness, we have used a spiral micrometer to measure the pellet thickness. Each one pellets has been measured five times on different places and then the average was calculated to gain the final value. It can be found that the thickness of the four samples were 1.294 mm, 1.288 mm, 1.231 mm, and 1.067 mm, respectively, which is well agreed with SEM results. Additionally, some small particles indexed as red circles can be detected in Fig. 2g; the small particles should be Sr and Co precipitation due to LSCF degradation as previously reported [19]. However, just a small amount of LSCF have been degraded in our case, because few particles can be observed in SEM image and no Sr and Co concerned peaks can be detected in the XRD patterns as Fig. 1 reveals.

**Fig. 3:**
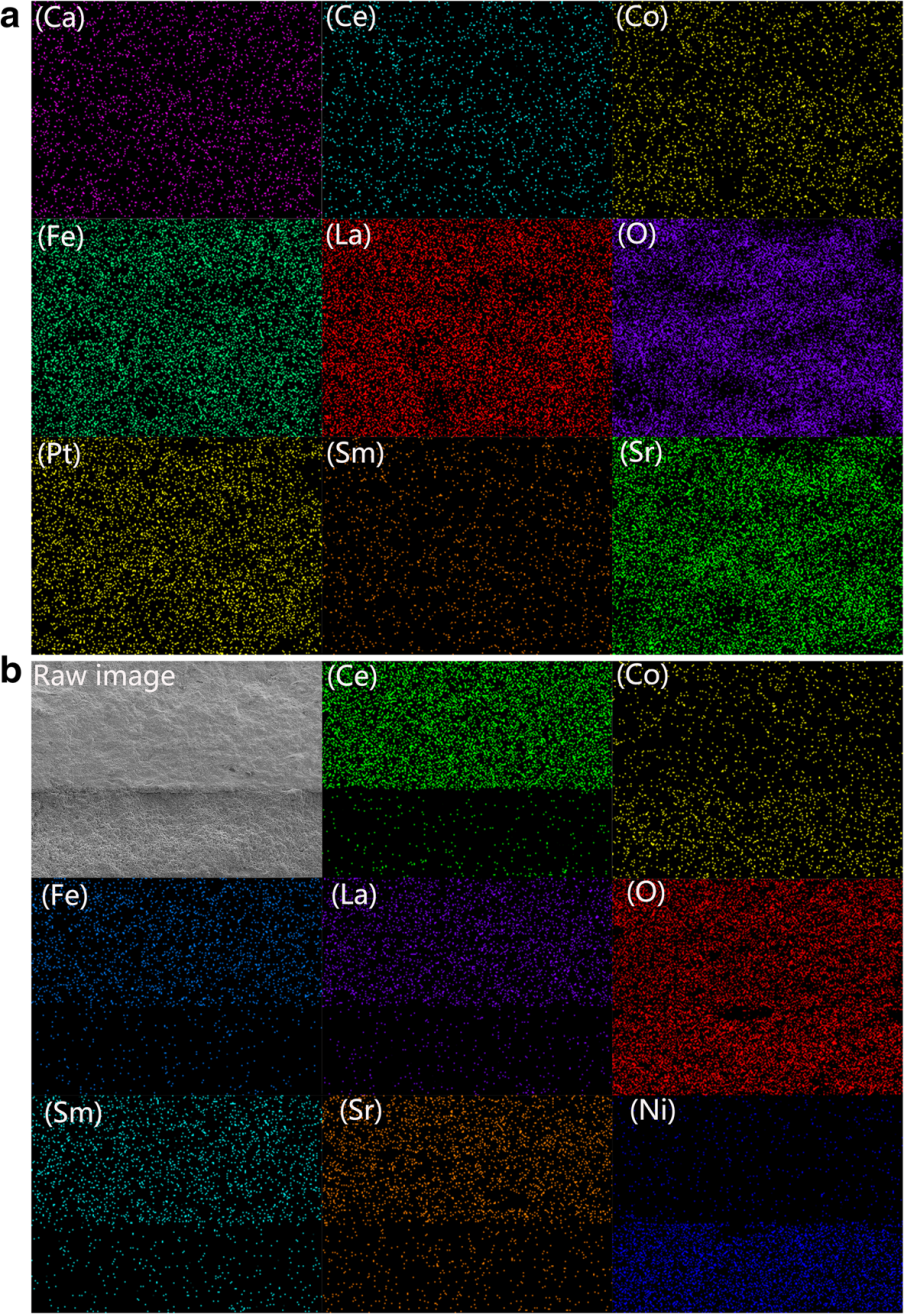
**a** EDS mapping images of the LSCF-SCDC pellet sintered at 1000 °C. **b** The cross-sectional SEM image and element mapping for the interface between LSCF-SCDC membrane and NCAl electrode

The EDS elemental mapping measurement was used as an implement to explore the element distribution in the agglomerate LSCF-SCDC pellet sintered at 1000 °C, as shown in Fig. 3a. It can be observed that Ca, Sm, and Ce elements derived from fluorite SCDC and Co, Fe, La, and Sr elements indexed as LaSrCoFe-oxide are evenly spread over the entire surface, indicating that although the LSCF-SCDC severely agglomerate after 1000 °C sintering, the element distribution remains uniform. The additional EDS mapping images were provided in Additional file 1. All the elements are homogeneously distributed on the cross-sectional surface of the four pellets, reflecting both the LSCF and SCDC phases were staying uniformly and formed homogeneous ceramic composite even at a long time sintering.

The detailed interface between LSCF-SCDC membrane and NCAL electrode after cell test has been provided in Fig. 3b. As the raw image shows, the NCAL electrode well contact with the LSCF-SCDC membrane and no obvious gap were observed at the interface; this may be due to the preheating treatment before performance testing. From the elemental mapping, the presence of Ce, Sm, Fe, La, Sr, Co, and O elements for the up layer confirmed the main component of LSCF-SCDC. Homogeneous distribution of Ni and Co can be observed in the down layer, indicating the electrode consisted of NiCo-oxide. The signal of Li element is too light to be detected, and the Al content in the NiCoAlLi-oxide (NCAL) layer is very low; therefore, very weak Al signal can be collected. It is worthily mentioned that no obvious elemental diffusion was found after cell operation. In addition, a heterogeneous gap was detected on the interface from the mapping images, which is mainly attributed to the damage during the scissoring of cross-sectional areas for SEM characterization.

### Fuel cell performances

**Fig. 4:**
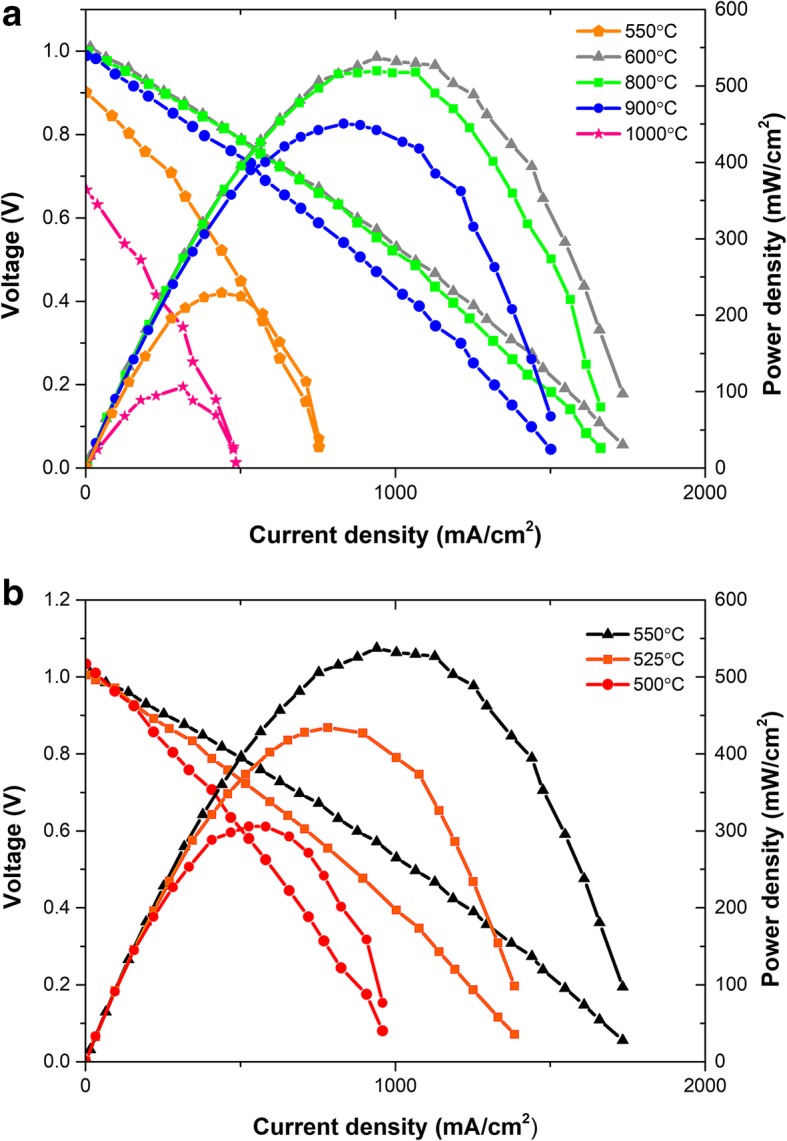
Voltage-current density and power density-current density characteristics for the **a** fuel cells assembled from LSCF-SCDC pellets sintering at various sintered temperatures. **b** The electrochemical performance of 600 °C sintered cell operating at 500–550 °C

The fuel cells have been fabricated using LSCF-SCDC powders sintered at different temperatures. These powders were used as ion-conducting membrane and Ni-foam NCAL as electrodes. In this work, as mentioned previously, the effect of sintering temperature toward the electrochemical performance is investigated. The typical current density (*I*)-voltage (*V*) and current density (*I*)-power density (*P*) curves for the fabricated fuel cells at 550 °C under H_2_/air supply are displayed in Fig. 4a. It can be seen that the device assembled by powders sintered at 600 °C has a maximum power density of 543 mW/cm^2^ and open circuit voltages (OCV) above 1 V. The results indicate the membrane of the pellets sintered at 600 °C is dense enough; otherwise, gas leakage will reduce the partial pressure of oxygen leading to a decrease of OCV according to Nernst equation. The underlying reason on how to avoid gas leakage at such low sintering temperature can be explained as the following: NCAL as electrodes should be reduced to metal Li, Ni, and Co in the anode side. The metal Li with strong activity should react with generated water to produce LiOH, which is in the molten state at the operational temperature and fully filled into the pores of SIMs to obtain dense pellet. Such a result will be reported in our next work. When the sintering temperatures increase to 800 °C or 900 °C, the corresponding performance reduced with some extent, but the OCV remained 1 V. For the fuel cell prepared by powders sintered at 1000 °C suffered significantly deteriorated, the OCV has reduced to lower than 0.7 V and the maximum power density dropped to 106 mW/cm^2^ simultaneously. The results reflect that the optimized sintering temperatures and microstructures influence directly the ionic transport and, in other words, portray the fuel cell performances. The high-temperature sintering leads to severe agglomeration which is already shown in the images of SEM in Fig. 2; as a result, the electrochemical performance of the assembled cells can be easily understood. At high temperatures, LSCF and SCDC particles can be melted to form eutectics; this gives rise to a high density of LSCF-SCDC pellet, accompanying with the big loss of the surfaces and interface contacts. This result is consistent with the previously reported data; Murray et al. revealed that LSCF becomes dense by sintering temperature higher than 1000 °C [24, 25]. The interfaces between LSCF and SCDC particles provide fast ion transport pathway, and it is an important factor for the ionic conduction of LSCF-SCDC pellets [26]; in this way, the so-called composite effect widely exists in two-phase or multi-phase materials [27, 28]. The high sintering temperature largely eliminates the interface area between LSCF and SCDC, and consequently, the ionic conducting pathways have been significantly reduced, finally leading to big losses in both OCV and power output. We have fabricated a fuel cell from LSCF-SCDC pellet sintered at 550 °C, and such device delivered an OCV of 0.9 V and maximum power density of 245 mW/cm^2^ at an operational temperature of 550 °C. The pregnant point is that the 600 °C fuel cell presents better performance than that of the 550 °C sample; it may be due to the porous structure of the LSCF-SCDC pellet when the sintering temperature is 550 °C, which may result in gas crossing over and short-circuiting that happened with some extent. In a sense, the specificity of the sintering temperature that has an influence on the performance of the pellet cannot be generalized. On the one hand, the higher sintering temperature should result in better density, accompanying superior cell performance. On the other hand, the higher sintering temperature should seriously destroy the interface between LSCF and SCDC two-phase materials, reducing the electrical conductivity to further deteriorate the cell performance. The two influences work together and reaches a balance, which leads to the optimal sintering temperature of 600 °C for cell performance.

The higher sintering temperature resulted in a thinner electrolyte membrane as the SEM result shown, which is the benefit for reducing the ohmic losses and should result in better cell performance. Oppositely, the cells based on higher temperature sintering delivered deteriorating cell performance. The only result for this phenomenon should be the superior ionic conductivity for low-temperature sintering sample. The linear particle of a polarization plot corresponds to ohmic polarization involving the ionic resistance in electrolyte and electronic resistance in electrodes [29, 30]. Since the high-conductive electrode NCAL was utilized in our case, we can deem that all the ohmic polarization is contributed by ionic resistance. That is to say that the ionic resistance of LSCF-SCDC pellet can be estimated from the slope of polarization curve at ohmic polarization region, and then the ionic conductivity can be deduced from the ionic resistance using pellet dimensions. By this way, the ionic conductivity of 600 °C and 1000 °C pellet are 0.229 and 0.076 S/cm at 550 °C, respectively. Obviously, the LSCF-SCDC pellet sintered at 600 °C possessed higher ionic conductivity than that of 1000 °C pellet, which resulted in the better electrochemical performance of the assembled fuel cell.

Figure 4 (b) presents the typical *I*-*V* and *I*-*P* characteristics at various temperatures for the device fabricated with the pellets sintered at 600 °C. As shown in Fig. 4b, the OCVs increase from 1.00 to 1.05 V when the operational temperature decline from 550 to 500 °C; this phenomenon can be explained by the Nernst equation, and the maximum power of 543 mW/cm^2^ is achieved at 550 °C. It is noteworthy that such fuel cell exhibited promising performance at low temperature (312 mW/cm^2^).

### EIS characterization

**Fig. 5:**
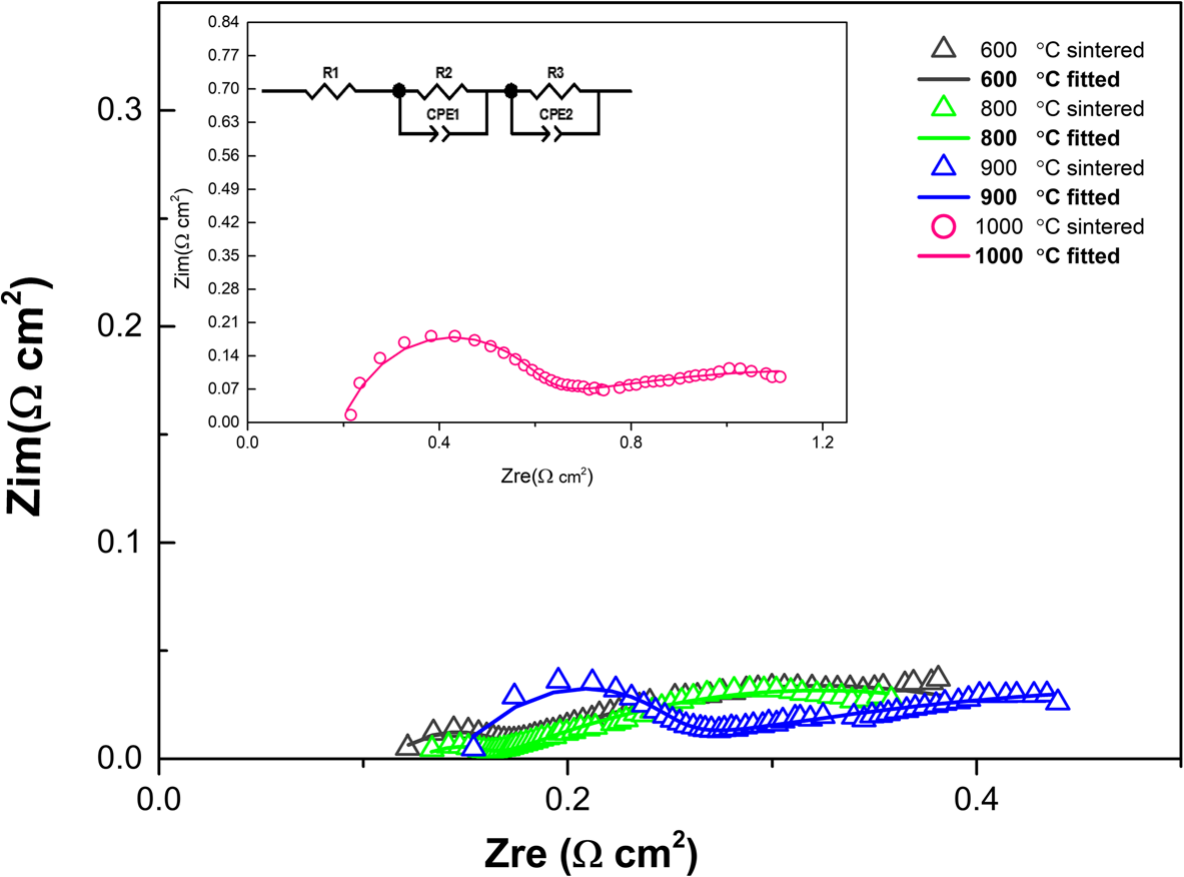
Impedance spectra of the LSCF-SCDC fuel cells with various sintered temperatures, tested in H_2_/air atmosphere at 550 °C. Point: primary Nyquist plots; Line: fitting Nyquist plots

To further study the electrochemical characteristics of these assembled cells, EIS measurements were conducted under H_2_/air condition, and the Nyquist curves recorded at different temperatures as shown in Fig. 5. All the spectra consist of depressed arc following a tail. The experimental data were fitted using ZSimpwin software. The corresponding equivalent circuit model *R*_1_(*R*_2_*Q*_2_)(*R*_3_*Q*_3_) is used to fit the measured data, where *R*_1_ is considered as ohmic resistance including the ionic transport resistance and the electron migration resistance. *R*_1_ is determined by the intercept of the real axis at high frequency. The sum of *R*_2_ and *R*_3_ are defined as the electrode polarization resistance (*R*p), which is closely related to the basic electrode reaction process, such as the oxygen molecule diffusion, adsorption, dissociation, and oxygen ion migration to triple phase boundary and incorporation into electrolyte processes during the oxygen reduction reaction. [31, 32]. The capacitance can be measured with the help of this relationship; ${C}_i=\frac{{\left({R}_i{Q}_i\right)}^{1/n}}{R_i}$

where *Q* is the constant phase element (CPE) and represents a non-ideal capacitor, *R*_*i*_ (*i* = 2.3) is the above resistance, and the associated *n* indicates the similarity of CPE to an ideal capacitor; when assuming *n* = 1, CPE can be deemed as an ideal capacitor [33, 34]. In the usual case, *n* is lower than 1. Each arc (*R*_i_*Q*_i_) (*i* = 2.3) should be attributed to corresponding processes according to the value of its characteristic capacitance *C*_*i*_. The fitting results are listed in Table 1. The ohmic resistances increase from 0.1112 to 0.2174 Ω cm^2^ corresponding to the sintering temperature from 600 to 1000 °C, respectively; this is because the high-frequency arc is dependent on the grain boundary resistance [35], which is strengthened by the agglomerate portion with sintering temperature increasing. *R*_2_ with a characteristic capacitance in the range of 10^−5^~10^−6^ F cm^−2^ for sintering temperature at 600 °C and 800 °C samples can be assigned to the ionic transfer reaction at the electrode/electrolyte interface. For sintering temperatures 900 °C- and 1000 °C-based fuel cells, the capacitance is 10^−7^~10^−8^ F cm^−2^; therefore, the *R*_2_ belongs to the grain boundary transfer process [36, 37]. The corresponding capacitance of *R*_3_ is higher than 10^−3^ F cm^−2^, indicating the *R*_3_ is contributed by both gas diffusion and charge transport processes. Compared with previous research [20], the ohmic resistance of these samples is within a normal level, but the polarization resistance reached as high as 1.2212 Ω cm^2^ when the intensified sintering temperature is 1000 °C.

**Table 1:** The fitting results of impedance spectra using the *R*_1_(*R*_2_*Q*_2_)(*R*_3_*Q*_3_) equivalent circuit. Resistance (*R*); *Q* has a unit of F cm^−2^

S. temp	*R*_1_	*Q*_1_	*n*	*C*_1_	*R*_2_	*Q*_2_	*n*	*R*_3_	*C*_2_
600 °C	0.1112	1.143	0.3026	9.848E−3	0.0603	2.483E−4	0.72	0.0291	3.847E−6
800 °C	0.1377	1.254	0.2856	1.55E−2	0.0280	2.677E−4	0.82	0.0339	2.409E−5
900 °C	0.1464	1.388	0.813	0.9620	0.1282	2.834E−4	0.47	0.5949	1.96E−8
1000 °C	0.2174	1.472	0.80	1.10713	0.4718	3.146E−4	0.57	0.7494	8.34E−7

### Electrical conductivity

**Fig. 6:**
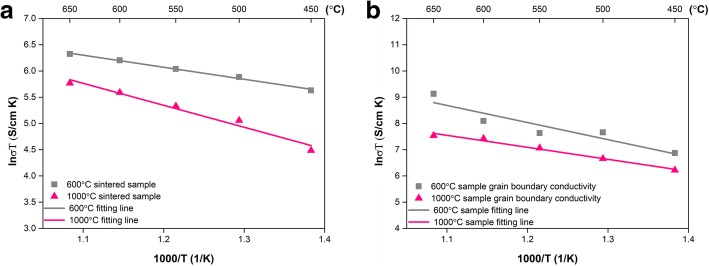
The Arrhenius plots of pellet sintered at 600 °C compared to that of pellet obtained at 1000 °C in **a** total conductivity and **b** grain boundary conductivity

To discuss the performance of assembled fuel cell from electrical conductivity dimensions, the pellets resistances are obtained from EIS results in the temperature range of 450–650 °C under air atmosphere. The bulk resistance (*R*_b_) is determined by the ion conducting within the grain bulk, and the resistance of grain boundaries (*R*_gb_) derives from the ionic conduction along or across the grain boundaries; both *R*_b_ and *R*_gb_ contribute to the total resistances of LSCF-SCDC pellets. Hence, the total conductivity *σ* at different temperatures can be obtained by the following formula: $\sigma =\frac{L}{R\times S}$

where *R* is the total resistance, and *L* and *S* are the thickness and the surface area of the pellets, respectively [38]. Arrhenius plots of pellets sintered at 600 °C and 1000 °C are shown in Fig. 6a. The Arrhenius curves show the linear relation from both samples disclosing that the mechanism of conduction does not change in the temperature range of 450–650 °C. The obtained *σ* for the pellet sintered at 600 °C starts from 0.3852 S/cm at 450 °C and reaches a maximum value of 0.6041 S/cm at 650 °C. The high conductivity should come from the bulk heterostructure between the two-phase materials LSCF and SCDC in the pellet as well the interface area between particles that form a space charge region and structural misfit, which promotes the ion conduction and results in good electrical conductivity. In addition, composite doping ceria with carbonate was considered as a typical strategy to receive ion-conducting enhancement [39, 40].

The recent investigation revealed that the semiconductor or SIMs have desired ionic conductivity [41, 42]. In our case, the LSCF-SCDC system is a SIM composite and the interface area between two constituent phases of the materials is responsible for the promising conductivity enhancement. However, such high conductivity sharply drops for the pellet sintered at 1000 °C, and the conductivity decrease should result from the increase of grain boundary resistance (*R*_b_), which is contributed by the ionic transport across or along the interface area. Such transport is closely related to the interface area as well as the particle size. The LSCF-SCDC pellets sintered at 1000 °C showed that the bulk structure and the interface area have been severely eliminated. Therefore, the pellets sintered at 1000 °C exhibited far lower values of 0.3463 S/cm at 650 °C and 0.1226S/cm at 450 °C in comparison with sintering temperature 600 °C. Moreover, the plots show that the activation energy of these samples are almost at the same level, and the activation energy 6.0711 kJ/mol for pellets sintered at 600 °C and the pellets sintered at 1000 °C pellet has 6.2060 kJ/mol. It shows that the activation energy has a weak correlation with the sintering temperature but has a greater relationship with the material itself.

The conducting mechanism in SIMs is very important for determining the electrochemical performance of the assembled fuel cell. Therefore, in our previous work, we have extensively investigated the interfacial conducting in SCDC-LSCF SIMs through STEM characterization combined with EELS [38]. It can be found that the depletion of oxygen vacancies inside the interface was significantly mitigated, which can be detected from the enrichment of oxygen in the LSCF-SCDC interface region as the EELS line scanning result presented, finally leading to the enhanced electrical conductivity for LSCF-SCDC SIMs in comparison with single phase materials. The similar phenomenon was observed in Ce_0.8_Gd_0.2_O_2-δ_-CoFe_2_O_4_ SIMs composite, where a Gd- and Fe-rich phase was in situ formed, which avoids the oxygen vacancy depletion in the grain boundary and resulted in enhancing grain boundary ionic conductivity [43].

The present work just peered the interfacial conducting mechanism from the effect of sintering temperature toward electrical conductivity. As Fig. 6a shows, the pellet sintered at 1000 °C delivered pretty lower electrical conductivity than that of the 600 °C pellet in all temperature ranges. The poor electrical conductivity for LSCF-SCDC pellets sintered at 1000 °C is attributed to its bulk structure, which can be observed from the SEM image. The bulk structure possessed few interface area between particles, which provided a high pathway for charge transfer. In other words, the pathway for charge conducting has been seriously destroyed when the sintering temperature reached as high as 1000 °C. The electrical conductivity combined with the SEM result provided direct and strong evidence for interfacial conducting.

In order to further verify interfacial conducting, we have specially separated the grain boundary resistance from the EIS results and converted the resistance to conductivity by using the pellet dimensions. The grain boundary conductivity (*σ*_gb_) as a function of temperature was presented as Fig. 6b. It can be found that *σ*_gb_ increased with temperature and the Arrhenius curves can be fitted by a single straight line. The noteworthy point is that the *σ*_gb_ of pellet sintered at 600 °C is higher than that of 1000 °C pellet. As we knew, the *σ*_gb_ is originated from the interface area, and the enhanced *σ*_gb_ of 600 °C pellet indicated superior interfacial conducting, proving the interfacial conducting mechanism in SIMs.

## Conclusion

We have characterized the morphology, microstructure, and electrical conductivity of LSCF-SCDC pellets sintered at different temperatures and successfully applied the SIM as an electrolyte to fabricate SOFC. As the electrochemical results revealed, when the sintering temperature increases from 600 to 1000 °C, the peak power density drops from 543 to 106 mW/cm^2^, and the OCVs decreased from 1.01 to 0.7 V simultaneously. The underlying reason for the deterioration could be the increase in ohmic resistance and severe polarization loss with the sintering temperature increasing gradually. As the SEM images show, high-temperature sintering significantly decreases the interface area between two phase materials, which can provide the ionic transport pathway. Through this work, it could simply be understood how sintering temperature affects ionic conduction. It is found that the interfacial ionic conduction plays a central role in the LSCF-SCDC SIMs’ electrical property and fuel cell device performances.

## Additional file

Additional file 1: **Figure S1.** The EDS mapping of 600 °C sintered pellet cross-section. **Figure S2.** The EDS mapping of 800 °C sintered pellet cross-section. **Figure S3.** The EDS mapping of 900 °C sintered pellet cross-section. **Figure S4.** The SEM images for pure LSCF and pure SCDC powder. **Figure S5.** The photo of measuring the pellet thickness by spiral micrometer. (DOC 8902 kb)

## Abbreviations

CPE: Constant phase element
EDS: Energy-dispersive spectrometer
EFFC: Electrolyte- free fuel cell
EIS: Electrochemical impedance spectroscopy
LSCF: La_0.6_Sr_0.4_Co_0.2_Fe_0.8_O_3-δ_
LT-SOFC: Low-temperature solid oxide fuel cell
NCAL: Ni_0.8_Co_0.15_Al_0.05_LiO_2-δ_
OCV: Open circuit voltage
*P*_max_: Peak power density
*R*_b_: Buck resistance
*R*_gb_: Resistance of grain boundaries
SCDC: Ce_0.8_Sm_0.05_Ca_0.15_O_2-δ_
SDC: Samarium-doped ceria
SEM: Scanning electron microscopy
SIM: Semiconductor-ionic material
XRD: X-ray diffraction
YSZ: Yttrium-stabilized zirconia
