# Supplementary material for: The sintering temperature effect on electrochemical properties of Ce0.8Sm0.05Ca0.15O2-δ (SCDC)-La0.6Sr0.4Co0.2Fe0.8O3-δ (LSCF) heterostructure pellet
Source: Nanoscale Res Lett. 2019 May 14;14:162. doi: 10.1186/s11671-019-2979-x (PMC6517467; doi:10.1186/s11671-019-2979-x)
Supplement: Supplementary file 1 — Figure S1. The EDS mapping of 600 °C sintered pellet cross-section. Figure S2. The EDS mapping of 800 °C sintered pellet cross-section. Figure S3. The EDS mapping of 900 °C sintered pellet cross-section. Figure S4. The SEM images for pure LSCF and pure SCDC powder. Figure S5. The photo of measuring the pellet thickness by spiral micrometer. (DOC 8902 kb) [file 11671_2019_2979_MOESM1_ESM.doc]

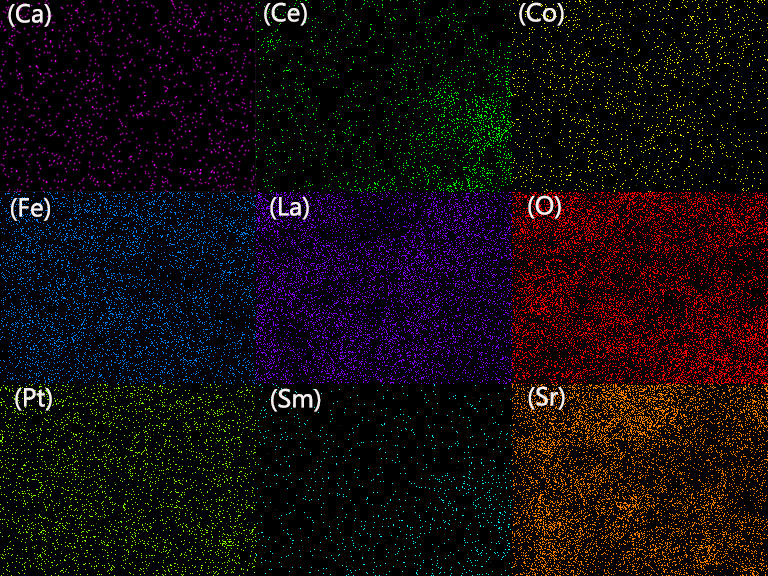


**Fig.S1** The EDS mapping of 600^o^C sintered pellet cross-section.


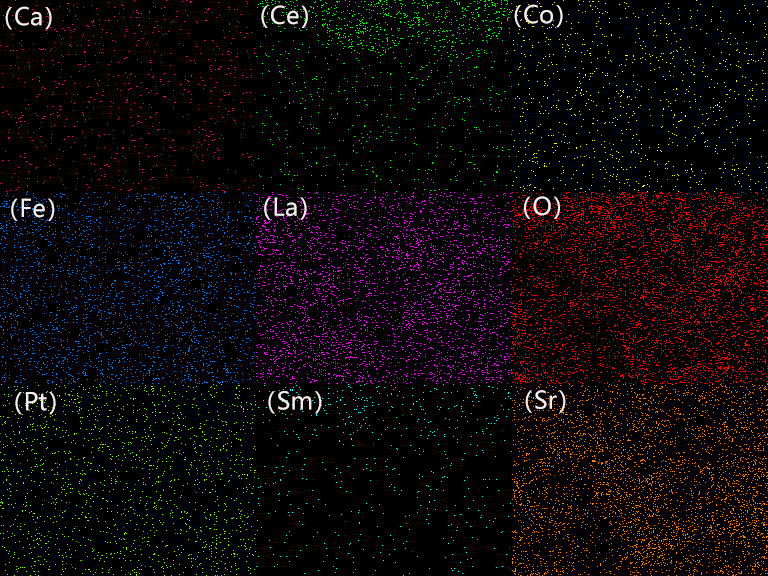


**Fig.S2** The EDS mapping of 800^o^C sintered pellet cross-section.


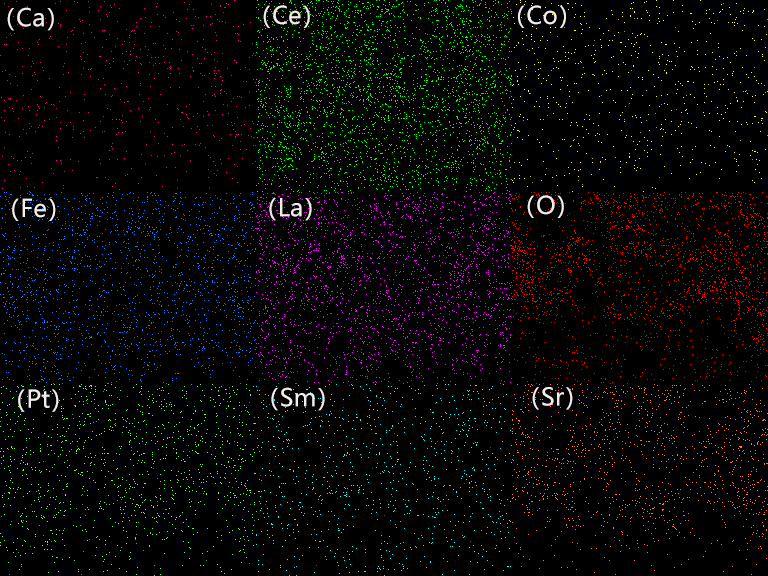


**Fig.S3** The EDS mapping of 900^o^C sintered pellet cross-section.


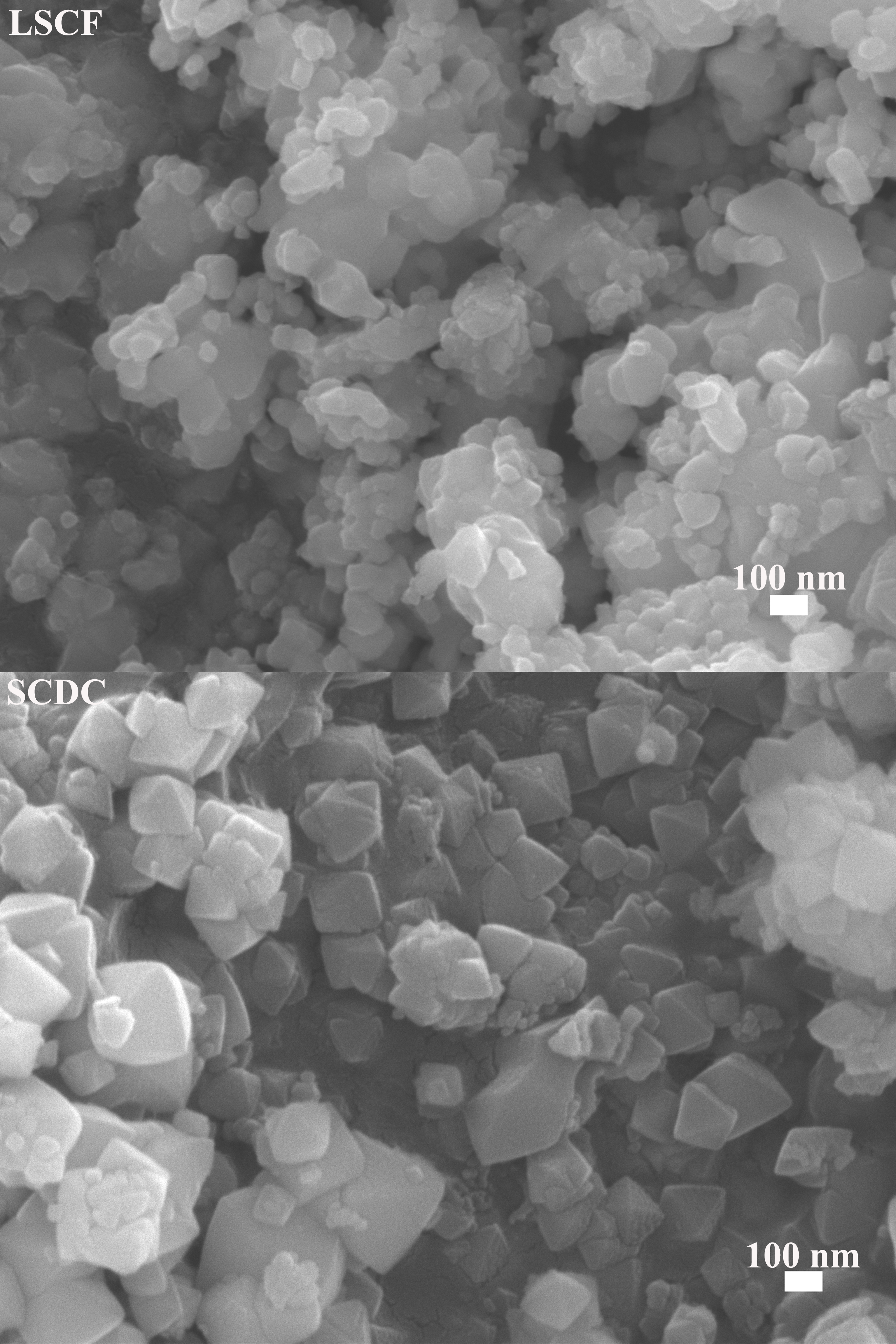


**Fig.S4** The SEM images for pure LSCF and pure SCDC powder.


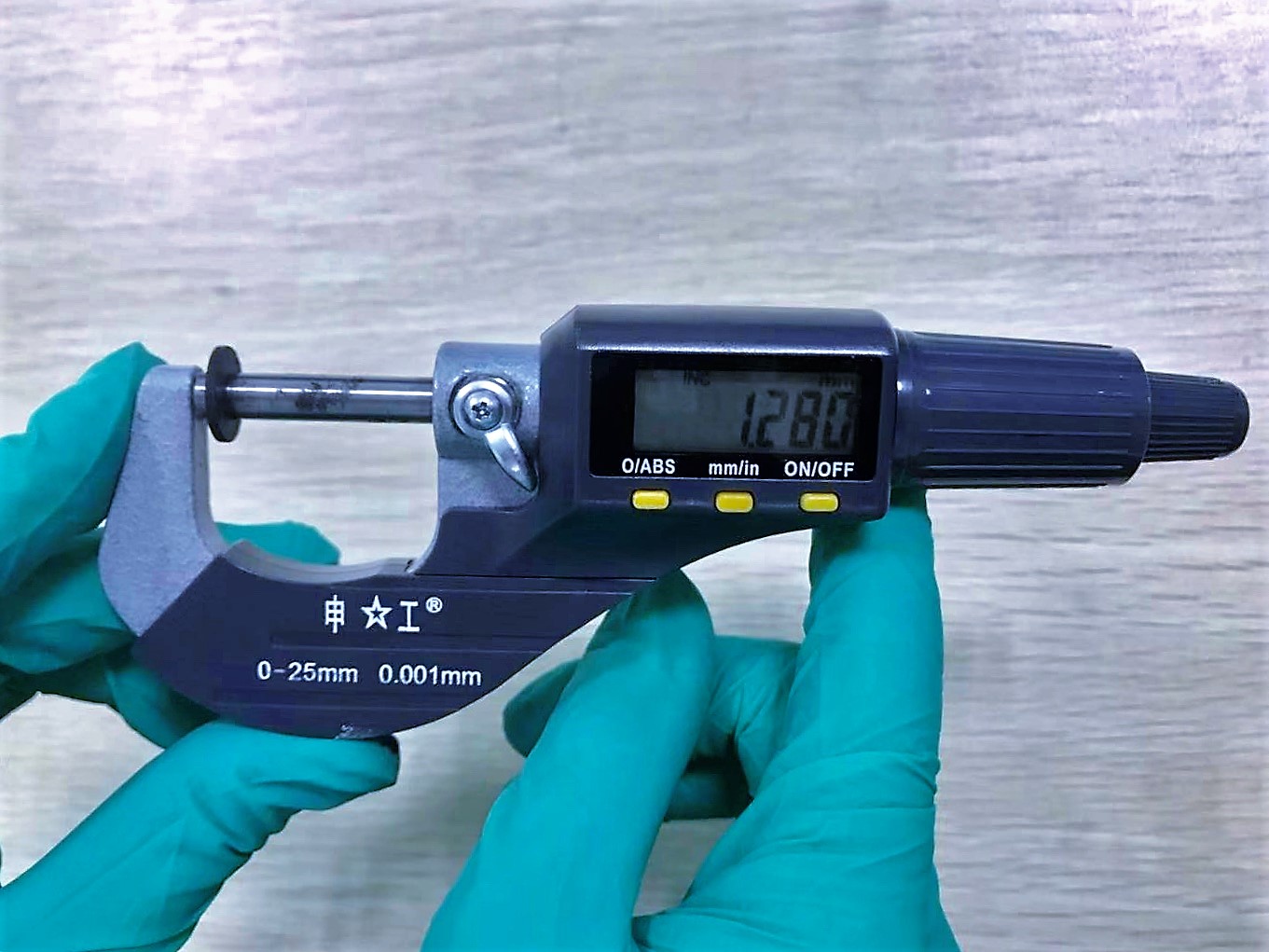


**Fig.S5** The photo of measuring the pellet thickness by spiral micrometer.
